# A bio-inspired feature extraction for robust speech recognition

**DOI:** 10.1186/2193-1801-3-651

**Published:** 2014-11-04

**Authors:** Youssef Zouhir, Kaïs Ouni

**Affiliations:** Research Unit: Signals and Mechatronic Systems, SMS, Higher School of Technology and Computer Science (ESTI), University of Carthage, Carthage, Tunisia

**Keywords:** Auditory filter model, Feature extraction, Hidden Markov Models, Noisy speech recognition

## Abstract

In this paper, a feature extraction method for robust speech recognition in noisy environments is proposed. The proposed method is motivated by a biologically inspired auditory model which simulates the outer/middle ear filtering by a low-pass filter and the spectral behaviour of the cochlea by the Gammachirp auditory filterbank (GcFB). The speech recognition performance of our method is tested on speech signals corrupted by real-world noises. The evaluation results show that the proposed method gives better recognition rates compared to the classic techniques such as Perceptual Linear Prediction (PLP), Linear Predictive Coding (LPC), Linear Prediction Cepstral coefficients (LPCC) and Mel Frequency Cepstral Coefficients (MFCC). The used recognition system is based on the Hidden Markov Models with continuous Gaussian Mixture densities (HMM-GM).

## Introduction

The Automatic speech recognition (ASR) system is one of the leading technologies acting on man–machine communication in real-world applications (Furui 2010). The ASR system is composed of two main modules. The first one is the acoustic Front-end (or feature extractor). This module generally uses the classical acoustic feature extraction techniques such as Perceptual Linear Prediction (PLP) (Hermansky 1990), Linear Prediction Coding (LPC) (Atal and Hanauer 1971), Linear Prediction Cepstral Coefficients (LPCC) (Atal 1974) and Mel Frequency Cepstral Coefficients (MFCC) (Davis and Mermelstein 1980). The second module is the classifier which is commonly based on the Hidden Markov Models.

The early feature based techniques involve incorporation of different psychoacoustic and neurophysical knowledge obtained from the study of the human auditory system which is capable of segmenting, localizing, and recognizing speech signal in noisy conditions without a noticeable degradation in performance of recognition (Rabiner and Juang 1993).

Generally, the feature extraction techniques are based on auditory filter modelling which uses a filterbank to simulate the cochlear filtering (Meddis et al. 2010). The efficient modelling of this auditory filterbank will improve the recognition performance and the features robustness in noisy environments.

The gammatone filterbank has been employed as the auditory filter modelling in various speech processing systems such as the Computational Auditory Scene Analysis system (Wang and Brown 2006).

Irino and Patterson have proposed an excellent candidate model for asymmetric, level-dependent cochlear filter called the Gammachirp auditory filter consistent with basic physiological data (Irino and Patterson 1997, 2006). This filter represents an extension of the gammatone filter characterized by an additional chirp parameter in order to produce an asymmetric amplitude spectrum. It provides an approximation of the auditory frequency response.

In this paper, we propose a biologically-inspired feature extraction method for robust recognition of noisy speech signals. The proposed method is based on the human auditory system characteristics, and relies on both the outer and middle ear filtering and the spectral behaviour of the cochlea. The outer and middle ear filtering is modelled by a second-order low-pass filter (Martens and Van Immerseel 1990; Van Immerseel and Martens 1992). The cochlear filter is modelled by a gammachirp auditory filterbank consisting of 34 filters, where the centre frequencies are equally spaced on the ERB-rate scale from 50 Hz to 8 kHz.

The HTK 3.4.1 toolkit is exploited in the Model training and recognition of speech signals. It is based on Gaussian Mixture density Hidden Markov models (Young et al. 2009). In our work, the HMM is trained for each word with five observation states and each state emission density consists of the four Gaussian Mixture densities.

The recognition performance of our feature extraction method was evaluated on speech signals corrupted by real-world noisy environments. The obtained results are compared to those obtained using PLP, LPC, LPCC and MFCC.

The paper is organized as follows: After introduction, section 2 presents the speech recognition system based on the hidden Markov models. It also introduces the classic feature extraction techniques of speech signals. In section 3, the proposed feature extraction method based on an auditory filter model is detailed, while introducing the auditory filter modelling. The experimental and evaluation results of our method are discussed in the section 4. Finally, conclusions are presented in the last section.

## The speech recognition system

**Figure 1:**
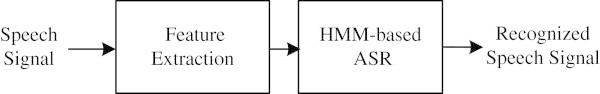
**Automatic speech recognition system.**

The process of the Automatic Speech Recognition system, as shown in Figure 1, can be divided into two main modules: feature extraction and HMM based ASR (Nadeu et al. 2001).

### The HMM based ASR

**Figure 2:**
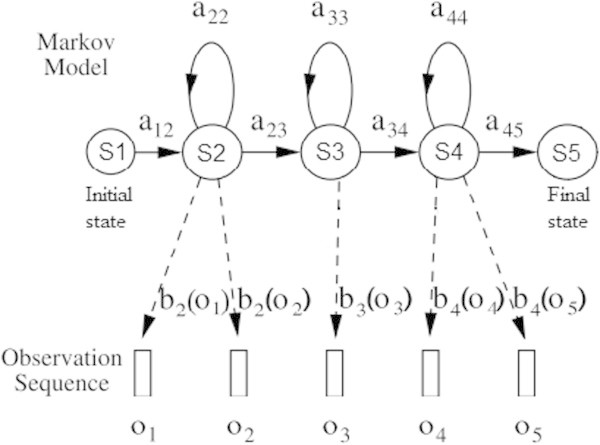
**The Markov Model with 5 states simple model (**Young et al. 2009 **).**

In HMM based ASR, the sequence of observed acoustic vectors (O = o_1_, o_2_, o_3_, …, o_t_, … o_T_, where o_t_ is the acoustic vector observed at time t) associated to each word is modelled as being generated by a Markov Model (Young et al. 2009) as shown in Figure 2.

The HMM represents a finite state machine which generates, at each state change, an acoustic vector *o*_*t*_ observed from the probability density *b*_*j*_(*o*_*t*_). The changes of state occur at every time unit according to the state transition probability from state *i* to state *j* is given by *a*_*ij*_. Figure 2 shows an example representing the observation sequence *o*_1_ to *o*_5_ for the state sequence S = 1, 2, 2, 3, 4, 4, 5, generated from a five state HMM with non-emitting entry and exit states. The HMM supports continuous Gaussian Mixture density distributions.

In the Gaussian Mixture density HMM, the probability distribution *b*_*j*_(*o*_*t*_) of being in state *j* at time *t* is given by (Young et al. 2009)
1

With *K*_*j*_ is the number of mixture components in state*j*, *c*_*jk*_ is the weight of the *k*’ th component and *N*(*o*; *μ*, ϑ) is a multivariate Gaussian defined by (Young et al. 2009)
2

Where *n* is the dimensionality of *o*, *ϑ* is covariance matrix and *μ* is mean vector.

### Classical feature extraction techniques

The most common techniques of feature extraction for speech recognition system employ the cepstral analysis to extract the feature coefficients from acoustic signal such as the MFCC and the LPCC. The MFCC technique consists to calculate the feature vectors from the frequency spectra at each frame of windowed speech. It is based on the human ear scale known the Mel scale.

The MFCC coefficients are calculated by applying a cosine transform to the real logarithm of short-term energy spectrum which has been expressed on a Mel-frequency scale.

The Linear Predictive Cepstral Coefficients (LPCC) is extracted from the speech signal by using the Linear Predictive Coding (LPC).
3

The Linear Predictive Coding (LPC) is based on the modelling of the vocal acoustic tract of human beings as a linear all-pole (IIR) filter defined by the following system function.
4

Where *p, G* and *a*_*k*_ are respectively the number of poles, the filter gain and the poles parameters which are called Linear Prediction Coefficients. The linear prediction coefficients are evaluated using the autocorrelation method.

**Figure 3:**
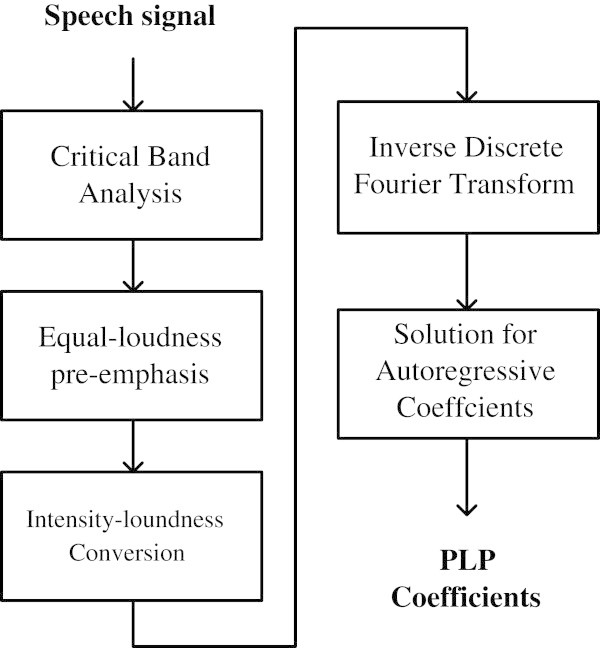
**Block diagram of PLP technique (**Hermansky 1990 **).**

The Perceptual Linear Prediction (PLP) is based on the human auditory system characteristics. It is similar to that of LPC technique, except that the speech power spectrum is transformed by a Bark-scale filter bank, an equal-loudness pre-emphasis and an intensity-loudness conversion to take into account the human auditory system characteristics, before modelling by the autoregressive all-pole transfer function. The block diagram of PLP technique, as shown in Figure 3 (Hermansky 1990; Beigi 2011).

## The proposed feature extraction based on an auditory filter model

The proposed extraction method of speech feature for ASR is based on an auditory filter model. This model simulates the outer/middle ear filtering and the spectral behaviour of the cochlea.

### Auditory filter modelling

The auditory filter modelling represents the mathematical model which tends to simulate the basic perceptual and psychophysical aspects of the human auditory characteristics (Lyon et al. 2010). This model consists of the simulation of the outer/middle ear filtering by second-order low-pass filter and the cochlea spectral behaviour by the gammachirp auditory filterbank.

The objective of outer/middle ear filtering is to increase the pressure of sound waves. This filtering is done by applying a low-pass filter that represents the sound transmission of outer/middle ear (Van Immerseel and Martens 1992). It is modelled by means of the transfer function given in Equation 5, transformed by means of a bilinear transformation and selecting a resonance frequency (*f*_*r*_ = 2*π*/*ω*_0_) equal to 4 kHz (Martens and Van Immerseel 1990; Van Immerseel and Martens 1992).
5

The gammachirp auditory filterbank simulates the signal processing in the cochlea, in particular it allows to obtain a good approximation of the basilar membrane frequency selectivity of the cochlear filter (Irino and Patterson 1997, 2006; Patterson et al. 2003). The Gammachirp filter represents an extension of the gammatone filter with the frequency modulation factor known as the chirp rate. Its analytic complex form is defined as (Irino and Patterson 1997).
6

Where time *t* > 0, *a*, *f*_0_, *φ* and *c* are the amplitude, the asymptotic frequency, the initial phase and the chirp rate respectively. *b* and *n* are the two parameters which define the gamma distribution envelope. “ln” denotes the natural logarithm.

The *ERB*(*f*_0_) is the equivalent rectangular bandwidth (*ERB*) of the Gammachirp auditory filters centered around *f*_0_ (Irino and Patterson 2006). The value of *ERB* is expressed by the following equation (Glasberg and Moore 1990; Moore 2012; Wang and Brown 2006).
7

The ERB-rate scale represents an approximately logarithmic function which relates the frequency value to the *ERBs* number, *ERBrate*(*f*), and can be expressed by (Glasberg and Moore 1990; Moore 2012; Wang and Brown 2006).
8

The Gammachirp Fourier spectrum is given by (Irino and Patterson 2006; Unokia et al. 2006).
9

Where  and *Γ*(n + jc) is the complex gamma distribution.

**Figure 4:**
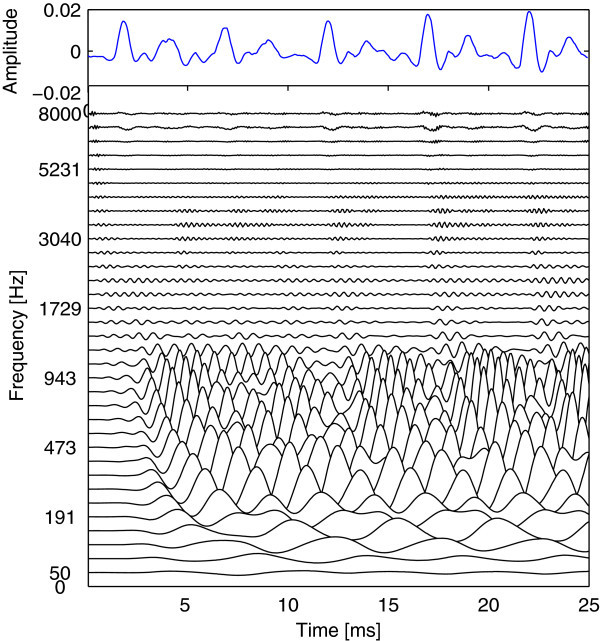
**The top panel represents the 25 ms waveform segment of the word “Water” (sampling frequency =16 kHz).** The bottom panel illustrates the simulation of BMM for the waveform segment.

The basilar membrane motion produced by a 34-channel Gammachirp auditory filterbank in response to a speech waveform segment is presented in Figure 4 (Bleeck et al. 2004). The waveform is the 25 ms of the word “Water” which is extracted from TIMIT database (Garofolo et al. 1990). The centre frequencies of the Gammachirp filters are equally spaced between 50 Hz and 8 kHz on the ERB-rate scale. Each individual line shows the output of one channel in the used auditory filterbank. The surface defined by the lines represents the simulation of basilar membrane motion (BMM). As illustrated in Figure 4, the concentrations of activity in channels above 191 Hz correspond to the resonance frequencies in the human vocal tract (Bleeck et al. 2004).

### The proposed feature extraction method

**Figure 5:**
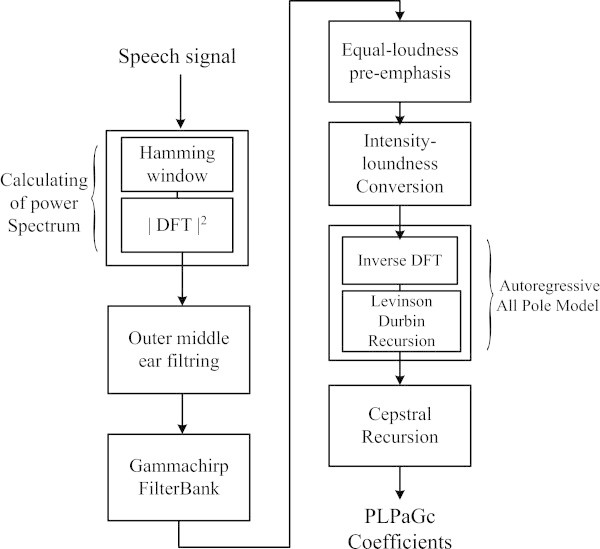
**Block diagram of the proposed Perceptual linear predictive auditory Gammachirp (PLPaGc) method.**

Our feature extraction method for speech recognition of noisy speech signal is based on auditory filter modelling. The proposed method, as illustrated by a block diagram in Figure 5, consists of seven steps. In the first step, the power spectrum is calculated by performing the square of Discrete Fourier Transform to the windowed segment of speech signal. The second step is the Outer and middle ear filtering, which is performed by a second order low-pass filter with a resonance frequency equal to 4 kHz (Martens and Van Immerseel 1990; Van Immerseel and Martens 1992). In the third step, the result is processed by applying the gammachirp auditory filterbank composed of 34 Gammachirp filters (Zouhir and Ouni 2013), where the centre frequencies of the filter are equally spaced in ERB-rate scale between 50 Hz and 8000 Hz (Glasberg and Moore 1990; Moore 2012). The output is pre-emphasized, in the fourth step, by the simulated equal loudness curve. The latter allows obtaining the non-equal sensitivity approximation of human auditory system at different frequencies (Hermansky 1990). The fifth step is the Intensity loudness Conversion step. The aim of this step consists in simulating the nonlinear relationship between the intensity of speech signal and perceived loudness by performing a cubic-root amplitude compression. In the sixth step, the autoregressive all-pole model is calculated using inverse DFT and the Levinson-Durbin recursion (Hermansky 1990). The last step of our method consists in applying a cepstral transformation to obtain the proposed Perceptual Linear Predictive Auditory Gammachirp coefficients (PLPaGc).

## Experimental results

This section evaluates the robustness of the proposed feature extraction method under various types of noisy environments.

### Databases and experimental setup

**Figure 6:**
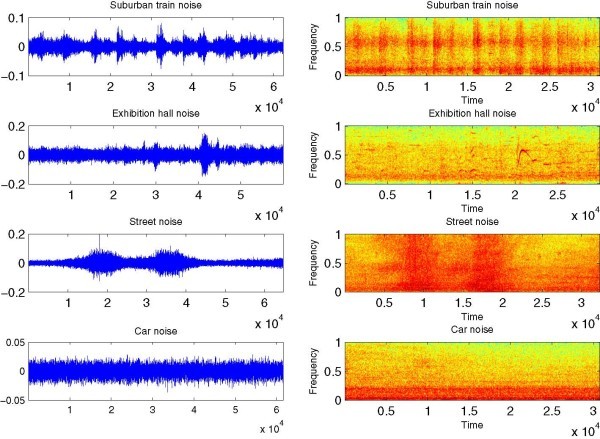
**The temporal representations and the spectrograms of the used noises.**

The TIMIT database (Garofolo et al. 1990) is used for all simulated speech recognition experiments. The used database is composed of speech signals sampled at 16 kHz of 630 speakers (female and male speakers) from 8 major dialect regions of the United States; each of them saying 10 sentences. We used isolated words extracted from this database. A total of 9702 isolated words were used in the training phase of the experiments and 3525 isolated words were used for the test phase. In order to evaluate the performance of our method on isolated words in the presence of various types of background noise, noisy corrupted tests sets were obtained by combining clean speech signals with suburban train, exhibition hall, street and car noises. These real-world noises were taken from AURORA database (Hirsch and Pearce 2000). Five noise levels, corresponding to 0 dB, 5 dB, 10 dB, 15 dB and 20 dB SNR values, where applied to each tests set. The temporal representations and the spectrograms of all used noises are shown in Figure 6.

The used speech recognition system is based on Hidden Markov Models. Our system employs the HTK 3.4.1 (Young et al. 2009) in the recognition task. The HTK 3.4.1 is a portable toolkit which allows the construction and manipulation of HMM-GM.

The HMM topology used in our experiments is a five states left-to-right model with a four Gaussian Mixture observation probability density distribution characterized by a diagonal covariance matrix.

The Table 1 represents the parameters of the Gammachirp function used in Gammachirp Auditory Filter.

**Table 1:** **Used Gammachirp parameters**

Parameter	Value
n	4
a	1
b	1.019
c	2
*φ*	0

### Results and discussion

For the baseline experiments, 12 coefficients of each technique were calculated from speech signal using Hamming analysis window with length equal to 25 ms and shifted with 10 ms steps.

The recognition performance of our feature extraction method has been compared to that of the classic techniques such as PLP, LPCC, LPC, and MFCC. The feature coefficients of each technique are combined with energy (*E*), differential coefficients first (∆) and second order (*A*) (12 coefficients +*E* + ∆ + *A*).

The experimental results obtained using the proposed PLPaGc feature and PLP, LPCC, LPC and MFCC feature in the noisy environments are summarized in the Tables 2, 3, 4 and 5. Four different noise types noise (suburban train, exhibition hall, street and car noises) with five noise levels (SNR) are considered.

**Table 2:** **Recognition rate (%) obtained by proposed and standard methods with suburban train noise**

	Recognition rate with HMM-4-GM					
	SNR level	PLPaGc	PLP	LPCC	LPC	MFCC
	0 dB	38.55	27.77	21.79	11.86	26.95
	5 dB	65.59	50.16	40.48	13.62	49.42
Suburban train noise	10 dB	84.71	72.74	60.96	18.47	71.66
	15 dB	92.74	85.82	77.90	28.96	86.30
	20 dB	95.77	91.72	87.06	41.96	92.60
	Average	75.47	65.64	57.64	22.97	65.39

**Table 3:** **Recognition rate (%) obtained by proposed and standard methods with exhibition hall noise**

	Recognition rate with HMM-4-GM					
	SNR level	PLPaGc	PLP	LPCC	LPC	MFCC
	0 dB	37.53	26.67	18.33	8.31	26.04
	5 dB	61.36	48.31	39.06	14.67	47.18
Exhibition hall noise	10 dB	81.73	69.30	60.54	20.65	68.74
	15 dB	90.58	84.17	77.99	29.87	84.09
	20 dB	95.74	91.40	86.92	40.00	92.14
	Average	73.39	63.97	56.57	22.70	63.64

**Table 4:** **Recognition rate (%) obtained by proposed and standard methods with street noise**

	Recognition rate with HMM-4-GM					
	SNR level	PLPaGc	PLP	LPCC	LPC	MFCC
	0 dB	39.86	32.03	25.13	10.52	30.64
	5 dB	65.90	51.60	41.73	12.65	50.52
Street noise	10 dB	84.26	72.99	60.51	16.88	73.13
	15 dB	92.84	85.93	76.79	26.35	86.33
	20 dB	96.00	91.63	87.09	38.04	92.31
	Average	75.70	66.84	58.25	20.89	66.59

**Table 5:** **Recognition rate (%) obtained by proposed and standard methods with car noise**

	Recognition rate with HMM-4-GM					
	SNR level	PLPaGc	PLP	LPCC	LPC	MFCC
	0 dB	45.96	28.51	23.15	10.13	29.19
	5 dB	70.81	56.37	46.55	13.50	56.14
Car noise	10 dB	88.94	80.57	70.87	20.65	81.08
	15 dB	94.84	91.55	86.07	31.74	92.23
	20 dB	96.74	94.89	91.60	43.21	95.63
	Average	79.46	70.38	63.65	23.85	70.85

As illustrated in the tables, the PLPaGc feature outperforms the four classic features in all noise conditions. For example, in the case of suburban train noise, the average of all noise levels of recognition rates achieved using PLPaGc feature is 75.47, while PLP, LPCC, LPC and MFCC feature provides respectively 65.64, 57.64, 22.97 and 65.39. It can be also observed that the recognition rates increase in all features when the noise level is decreased with respect to the signal level (i.e., SNR increases from 0 dB to 20 dB).

## Conclusion

A new auditory filter modelling-based feature extraction method for noisy speech recognition was presented in this paper. The proposed method was motivated by the research studies of the human peripheral auditory modelling. The used auditory model consists of simulating the outer/middle ear filtering by a second order low-pass filter and the cochlea spectral behaviour by the gammachirp auditory filterbank, where the values of those centre frequencies are chosen according to the ERB rate scale. The robustness of the proposed PLPaGc feature was evaluated on speech recognition rate in real-world noisy environments. The experimental results show that the PLPaGc feature gives better recognition rates compared to four classical PLP, LPCC, LPC and MFCC feature.

## References

[CR1] Atal BS (1974). Effectiveness of linear prediction characteristics of the speech wave for automatic speaker identification and verification. J Acoust Soc Am.

[CR2] Atal BS, Hanauer SL (1971). Speech analysis and synthesis by linear prediction of the speech wave. J Acoust Soc Am.

[CR3] Beigi H (2011). Fundamentals of Speaker Recognition.

[CR4] Bleeck S, Ives T, Patterson RD (2004). Aim-mat: the auditry image model in MATLAB. Acta Acustica United Ac.

[CR5] Davis SB, Mermelstein P (1980). Comparison of parametric representations for monosyllabic word recognition in continuously spoken sentences. IEEE Trans Acoust, Speech, Signal Processing.

[CR6] Furui S, Chen F, Jokinen K (2010). History and Development of Speech Recognition. Speech Technology.

[CR7] Garofolo J, Lamel L, Fisher W, Fiscus J, Pallett D, Dahlgren N (1990). DARPA, TIMIT Acoustic-Phonetic Continuous Speech Corpus CD-ROM. National Institute of Standards and Technology.

[CR8] Glasberg BR, Moore BCJ (1990). Derivation of auditory filter shapes from notched-noise data. Hear Res.

[CR9] Hermansky H (1990). Perceptual linear predictive (PLP) analysis of speech. J Acoust Soc Am.

[CR10] Hirsch H, Pearce D (2000). The Aurora Experimental Framework for the Performance Evaluation of Speech Recognition Systems Under Noisy Conditions.

[CR11] Irino T, Patterson RD (1997). A time-domain, level-dependent auditory filter: the Gammachirp. J Acoust Soc Am.

[CR12] Irino T, Patterson RD (2006). A dynamic compressive gammachirp auditory filterbank. IEEE Trans Audio Speech Lang Processing.

[CR13] Lyon RF, Katsiamis AG, Drakakis EM (2010). History and future of auditory filter models. Proceedings of 2010 IEEE International Symposium on Circuits and Systems (ISCAS).

[CR14] Martens JP, Van Immerseel L (1990). An auditory model based on the analysis of envelope patterns. Int Conf Acoust Speech Signal Process.

[CR15] Meddis R, Lopez-Poveda EA, Fay RR, Popper AN (2010). Computational Models of the Auditory System. Vol. 35.

[CR16] Moore BCJ (2012). An Introduction to the Psychology of Hearing.

[CR17] Nadeu C, Macho D, Hernando J (2001). Time Frequency and filtering of filter-bank energies for robust HMM speech recognition. Speech Comm.

[CR18] Patterson RD, Unoki M, Irino T (2003). Extending the domain of centre frequencies for the compressive gammachirp auditory filter. J Acoust Soc Am.

[CR19] Rabiner L, Juang BH (1993). Fundamentals of Speech Recognition. Prentice Hall Signal Processing Series.

[CR20] Unokia M, Irino T, Glasberg B, Moore BCJ, Patterson RD (2006). Comparison of the roex and gammachirp filters as representations of the auditory filter. J Acoust Soc Am.

[CR21] Van Immerseel LM, Martens JP (1992). Pitch and voiced/unvoiced determination with an auditory model. J Acoust Soc Am.

[CR22] Wang DL, Brown GJ (2006). Principles, Computational Auditory Scene Analysis: Algorithms, and Applications.

[CR23] Young S, Evermann G, Gales M, Hain T, Kershaw D, Liu X, Moore G, Odell J, Ollason D, Povey D, Valtchev V, Woodland P (2009). The HTK Book (for HTK Version 3.4.1).

[CR24] Zouhir Y, Ouni K, Drugman T, Dutoit T (2013). Speech Signals Parameterization Based on Auditory Filter Modelling. Advances in Nonlinear Speech Processing LNAI 7911, NOLISP 2013, Mons, Belgium.

